# Fatty Acid and Associated Gene Expression Analyses of Three Tree Peony Species Reveal Key Genes for α-Linolenic Acid Synthesis in Seeds

**DOI:** 10.3389/fpls.2018.00106

**Published:** 2018-02-05

**Authors:** Qing-Yu Zhang, Rui Yu, Li-Hang Xie, Md Mahbubur Rahman, Aruna Kilaru, Li-Xin Niu, Yan-Long Zhang

**Affiliations:** ^1^College of Landscape Architecture and Arts, Northwest A&F University, Xianyang, China; ^2^Department of Biological Sciences, East Tennessee State University, Johnson City, TN, United States

**Keywords:** alpha-linolenic acid, edible oil, fatty acid biosynthesis, fatty acid desaturases, omega-3, peony species, seed oil

## Abstract

The increasing demand for healthy edible oil has generated the need to identify promising oil crops. Tree peony (*Paeonia* section *Moutan* DC.) is a woody oil crop with α-linolenic acid (ALA) contributing for 45% of the total fatty acid (FA) content in seeds. Molecular and genetic differences that contribute to varied FA content and composition among the wild peony species are, however, poorly understood. Analyses of FA content and composition during seed development in three tree peony species (*Paeonia rockii*, *P. potaninii*, and *P. lutea*) showed varied FA content among them with highest in *P. rockii*, followed by *P. potaninii*, and *P. lutea*. Total FA content among these species increased with seed development and reached its maximum in its final stage. Seed FA composition analysis of the three species also revealed that ALA (C18:3) was the most abundant, followed by oleic (C18:1) and linoleic (C18:2) acids. Additionally, quantitative real-time RT-PCR analyses of 10 key seed oil synthesis genes in the three tree peony species revealed that *FAD3*, *FAD2*, β*-PDHC*, *LPAAT*, and *Oleosin* gene expression levels positively correlate with total FA content and rate of accumulation. Specifically, the abundance of *FAD3* transcripts in *P. rockii* compared with *P. potaninii*, and *P. lutea* suggests that FAD3 might play an important role in synthesis of ALA via phosphatidylcholine-derived pathway. Overall, comparative analyses of FA content and composition in three different peony species revealed a correlation between efficient lipid accumulation and lipid gene expression during seed development. Further characterization and metabolic engineering of these key genes from peonies will allow for subsequent improvement of tree peony oil quality and production.

## Introduction

Tree peony is a perennial deciduous shrub, belongs to section *Moutan* of the genus *Paeonia* in the family *Paeoniaceae* (Picerno et al., 2011; Li et al., 2012). Peonies are referred as ‘the king of flowers’ in China, where all nine wild species are endemic (Picerno et al., 2011; Yuan et al., 2011, 2012; Li et al., 2015a). Tree peonies in China have more than 2000 years of history (Zhou et al., 2014); they are cultivated in more than 20,267 hectares in hilly regions of China and their annual seed production exceeds 57,855 tons (Li et al., 2015b). Peony seeds are a good source of edible oil with higher percentage of unsaturated FA (92%). Specifically, α-linolenic acid (ALA/18:3), an ω-3 PUFA is the most predominant (∼45% of the total FA content) FA in peony seed, 18:3 (Li et al., 2015a,b).

Linoleic acid (18:2), an ω-6 FA and ALA are essential for humans as they cannot synthesize these two FAs and must be obtained from diet (Zhou et al., 2014). A lower percentage of ω-3 FA combined with a high percentage of ω-6 FA in the human diet has been implicated in chronic diseases such as diabetes, inflammatory and cardiovascular diseases, whereas increased levels of ω-3 FA (a high ω-3: ω-6 ratio) exert suppressive effects (Harbige, 2003; Bhunia et al., 2016). In plants, the proportion of LA to ALA varies significantly among different oilseed crops. For example, in most common edible oils derived from peanut, corn, olive, sunflower, sesame and camellia have less than 3% of 18:3 (Lee et al., 1998; Simopoulos, 2001; Bhunia et al., 2016). Notably, in tree peony seed oil while 18:3 accounts for ∼45% of the total FA content, 18:2 is less than 25% (Li et al., 2015a,b). With the exception of flax and perilla seed oils (Su et al., 2016), such high levels of ω-3 FAs are uncommon in oil crops. Tree peony, especially being a woody oil crop is therefore an attractive model for dissecting PUFA metabolic pathways and develop an alternative source of healthy edible oil.

Fatty acid biosynthesis and the mechanisms of triacylglycerol (TAG/oil) accumulation in seeds involve multiple subcellular organelles and require extensive lipid trafficking (Bates et al., 2013). Within developing seeds of oil crops, FA biosynthesis mainly occurs in plastids, which are subsequently exported to ER in the form of acyl-Coenzyme A (acyl-CoA) of mostly oleic acid (18:1), along with minor amounts of palmitic (16:0) and stearic (18:0) acids (Rawsthorne, 2002; Weselake et al., 2009; Troncoso-Ponce et al., 2011). Acyl-CoAs are typically utilized in the acylation of G3P or further elongated and/or desaturated in ER through acyl editing mechanism. In many oil crop seeds, most of the 18:1 enters into *sn*-2 position of glycerol backbone of membrane lipids as PC, where it can be further desaturated to 18:2 or 18:3 with the addition of double bonds catalyzed by ER-localized FA desaturases (FAD2 and FAD3). These PUFAs from PC can be hydrolyzed and incorporated into *sn*-3 position of DAG to form TAG by phospholipid diacylglycerol acyltransferase (PDAT). This pathway is referred to as acyl-CoA independent pathway, which is one of the major pathways for TAG biosynthesis in plants (Wickramarathna et al., 2015). Additionally, TAG can be synthesized through Kennedy pathway in an acyl-CoA dependent manner. In this process, the *de novo* assembly of TAG from G3P and acyl-CoAs involves three sequential acylations where the first two acylations of G3P are catalyzed by GPAT and LPAAT, respectively, followed by dephosphorylation by PAP to produce DAG; the last and final acylation is catalyzed by DGAT to produce TAG. The preferred pathway for TAG synthesis differs among plant species ranging from an acyl-CoA dependent Kennedy pathway to an acyl-CoA independent pathway (Bourgis et al., 2011; Troncoso-Ponce et al., 2011); in seeds, however, more than 90% FAs flux through PC before entering into TAG (Vrinten et al., 2005; Bates et al., 2013). More recently, additional pathways that include monoacylglycerol intermediates contributing to TAG synthesis were also proposed (El Tahchy et al., 2015; Simpson and Ohlrogge, 2016). Newly synthesized TAGs in seeds are typically stored within LD that are stabilized by LD-associated proteins such as oleosins and caleosins (Murphy, 1993; Murphy and Vance, 1999; Winichayakul et al., 2013). Additional or alternate proteins may also be associated with stabilization of LD in non-seed oil-accumulating tissues such as mesocarps of avocado and oil palm (Gidda et al., 2013; Horn et al., 2013). Although several oil biosynthesis genes are cloned and characterized and competing pathways are established for some oil crops (Rahman et al., 2016), the underlying molecular mechanisms of PUFA biosynthesis and accumulation such as that of 18:3 are poorly understood. Together, these studies suggest continual need for exploration and identification of preferred TAG biosynthetic pathways in diverse species for increasing the productivity of desired oil composition.

The use of select genes from wild relatives of crop plants to improve productivity is well practiced for more than 60 years (Hajjar and Hodgkin, 2007). Interestingly, FA composition and content vary significantly among different wild tree peony species suggesting a differentially regulated FA and/or TAG synthesis pathways in developing seeds. Previously, a transcriptome study of tree peony seeds (*Paeonia* section *Moutan* DC.) revealed *FAD2* and *FAD8* might play a role in FA synthesis and TAG accumulation (Li et al., 2015a). Here, a comparative approach was taken to associate differences in FA accumulation among three wild relatives of tree peony species that varied in seed oil content from low to high (150.1–271.8 mg g^-1^), with transcript levels of select lipid genes during seed development. Such comparative analyses are expected to identify potential candidate genes that likely contribute to differential accumulation of PUFAs and thus provide molecular tools for further modification to yield more and diverse lipids in peonies and other agronomically relevant species.

## Materials and Methods

### Plant Material

Nine wild tree peony species (*Paeonia rockii*, *P. ostii*, *P. ludlowii*, *P. decomposita*, *P. qiui*, *P. potaninii*, *P. jishanensis*, *P. lutea*, and *P. delavayi*) were used in this study. Seeds of tree peonies were collected in 2015 from the Wild Tree Peony Germplasm Repository at Gansu Forestry Science and Technology Extension Station, China (36°03′N, 103°40′E). Repository is located at an average elevation of 1520 m, where the average annual rainfall is 327 mm and temperature is 10.3°C, with sunshine duration of 2446 h, and frost-free period of more than 180 days. Wild tree peony germplasm seeds were introduced to the repository and were cultivated under same environmental conditions for 13 years. We monitored their seed development process from pollination until maturation (the pods break open and the color of seeds turn black) from May to August 2015. Pods from nine wild tree peony species were collected at five sampling points (S1 to S5) from 0 to 100 days at 20 days intervals. Each analysis was conducted with three biological replicates and each replicate included pod samples from a mixture of three trees. For RNA extraction at a later time, a portion of the collected seeds from different developmental stages was flash frozen in liquid nitrogen and stored at -80°C. Remaining seeds were dried naturally at room temperature and stored in a brown dryer filled with nitrogen for more than 48 h, and were subsequently used for FA content and composition analysis.

### Chemicals and Standards

Fatty acids were named using the formula Cx:yΔnc, as described previously (Simopoulos, 2006). FAME of myristic acid (C14:0), palmitic acid (C16:0), palmitoleic acid (C16:1Δ9c), heptadecanoic acid (C17:0), stearic acid (C18:0), oleic acid (C18:1Δ9c), LA (C18:2Δ9c,12c), ALA (C18:3Δ9c,12c,15c), γ-linolenic acid (C18:3 D6c,9c,12c) and Supelco^®^ 37-component FAMEs Mix (C4–C24 unsaturated) were purchased from Sigma–Aldrich (St. Louis, MO, United States). HPLC-grade methanol, chloroform, n-hexane, and n-pentane were used in this study, which were purchased from Alltech Scientific (Chaoyang District, Beijing, China). n-hexane was used as a solvent to prepare standard stock solutions and dilutions. Heptadecanoate (C17:0) was used as an internal standard because it is generally not present in biological samples. All compounds and stock solutions were stored according to manufacturer’s protocol.

### Lipid Extraction

Total lipids were extracted as described previously (Li et al., 2015b), with some modifications. Briefly, dried tree peony seeds were pulverized in liquid nitrogen using a Tissuelyser-24 (Jingxin Limited Company, Shanghai, China). Fifty milligrams of seed powder was extracted with 1.0 mL chloroform–methanol (1:2, v/v) and C17:0 was added as an internal standard (0.5 mg mL^-1^). Samples were homogenized with a Vortex Mixer S8223 (American Scientific Products, Lakewood, WA, United States) and kept at 4°C for overnight. Subsequently, 0.6 mL KCl (1 M) was added to make the final ratio of chloroform: methano1: KCl to 1:1:0.9. The mixture was vortexed vigorously and centrifuged at 10,000 rpm for 10 min. After centrifugation, the lower chloroform phase with lipids was collected and dried with a Savant SPD111V SpeedVac (Thermo Fisher Scientific, Waltham, MA, United States). FAs in total lipids were derivatized to methyl esters.

### Preparation of Fatty Acid Methyl Esters (FAMEs)

The dried FAs were methylated as previously described (Li et al., 2015b), using methanol-sulphuric acid as a methylating reagent. Briefly, 0.5 mL methanol containing 5% concentrated sulphuric acid was used to resuspend dried lipids by 1 h incubation in a 90°C water bath (Thermomix, Eppendorf, Hamburg, Germany). After cooling to room temperature, 0.5 mL of deionized water was added to terminate the derivatization reaction. FAMEs were subsequently extracted with 0.75 mL of n-pentane, centrifuged (10,000 rpm for 10 min) and the upper phase was then collected and dried with a speed vacuum as mentioned above. The dried pellet was resuspended in 200 μL n-hexane and 1 μL was injected for GC-MS analysis.

### Fatty Acid Analysis by GC–MS

Quantitative FA analysis was carried out using gas chromatography coupled with mass spectrometry (GC7890A/MS5975C, Agilent Technologies, Waldbronn, Germany). The GC was equipped with a G4513A autosampler and HP-88 column (100 m × 0.25 mm i.d., 0.20 μm film thickness). Ultra-high purity helium was used as carrier gas at a flow rate of 1.0 mL min^-1^ and analyses were performed in constant flow mode. The temperature of the transfer line, ion source, and quadrupole were set at 280, 230, and 150°C, respectively, while the injector temperature was set at 250°C for split injection at a split ratio of 10:1. The initial oven temperature was maintained at 120°C for 1 min before it was increased by 10°C min^-1^ to 175°C and kept isothermal for 10 min, and then at 5°C min^-1^ the temperature was ramped to 210°C for 5 min and 230°C for 5 min with a total run time is 37.5 min. Qualitative FA analysis was achieved by comparing the mass spectra to those available in the database (NIST08 Library) and co-elution with corresponding standards. A standard curve method with an internal standard was used as a quantitative approach to construct five calibration plots of analyte/internal standard peak-area ratio vs. standard concentration, as determined by the least squares method. FAMEs in each sample were measured using methyl heptadecanoate as the internal standard and expressed as milligrams per gram DW of a sample. All samples were analyzed in triplicates.

### Hierarchical Cluster Analysis

Total FAs and the content of five major FAs in seeds of nine wild tree peony species (**Supplementary Table S1**) were subjected to a cluster analysis using cluster program SPSS 17.0 (version 17.0 for Windows; SPSS Inc., 2008). A dendrogram was generated from the cluster analysis using Ward’s method. The members of the same cluster indicate relatively similar FA content and composition.

### Total RNA Extraction and Quantitative Real-Time RT-PCR

Total RNA was extracted from tree peony seeds using TIANGEN RNA Prep Pure Plant kit (Tiangen Biotech Co. Ltd., Beijing, China), and the quality and quantity of RNA were determined by Nanodrop and agarose gel electrophoresis. The first-strand cDNA was synthesized using PrimeScript^®^ RT reagent Kit with gDNA Eraser (DRR047A, Takara, Dalian, China) according to the manufacturer’s instructions. The qRT-PCR was performed using a SYBR^®^ Premix Ex Taq^TM^ kit (DRR041A, Takara, Dalian, China) on a LightCycler480 Real-Time PCR System (Roche Diagnostics, Basel, Switzerland). The temperature cycle of qRT-PCR reaction was as follows: 95°C for 15 s, followed by 40 cycles of 95°C for 5 s, 58°C for 30 s, and 72°C for 31 s. The fluorescence data were analyzed with LightCycler480 analysis software during the 72°C extension step. All reactions were carried out in triplicates. A wide range of reference genes including actin (ACT), tubulin, polyubiquitin (UBQ), glyceraldehyde-3-phosphate dehydrogenase (GAPDH), elongation factor-1α, and ribosomal genes are used in plants and animals to normalize the qRT-PCR data (Goidin et al., 2001; Bustin, 2002; Kim et al., 2003; Dheda et al., 2004; Li et al., 2015a). Here we tested ACT, UBQ, GAPDH2, and 18S-26S internal transcribed spacer (ITS) in different tissues of several tree peony species and 18S–26S ITS was selected as the best candidate, based on its stable expression profile (data not shown). Gene specific primers used in this study for select genes (**Supplementary Table S3**) were described previously (Li et al., 2015a). For qRT-PCR data analysis, expression values (*C*t) of select genes were normalized to 18S–26S ITS transcript levels, and 2^-ΔΔ^*^C^*^t^ values were shown as relative expression to transcript levels of S1 within each species (**Supplementary Figure S1** and **Supplementary Table S4**) and 20 DAP of *P. rockii* (**Figure 5**). Transcript abundance was quantified relative to S1 of *P. rockii* in order to observe the differences in gene expression level between the three species at the same developmental stage, especially at the initial stage when oil synthesis is at its lowest (**Figures 2B,C**).

### Statistical Analysis

All experiments included three biological replicates and technical replicates as previously indicated. Mean ± SD were determined and one-way analysis of variance was carried out using SPSS (version 17.0 for Windows; SPSS Inc., 2008), to determine significance at *P*-value < 0.05.

## Results and Discussion

**FIGURE 1:**
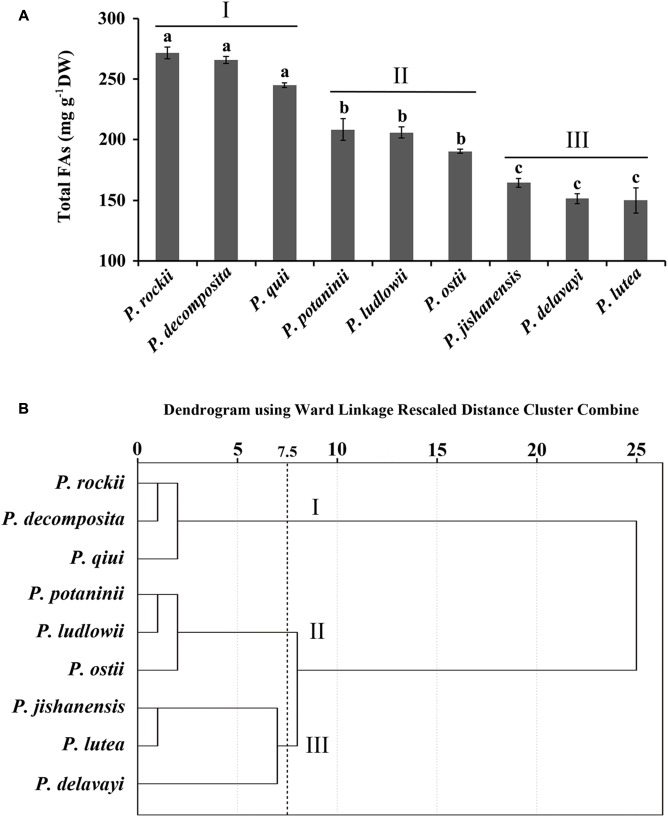
Association of nine tree peony species based on fatty acid content and composition. **(A)** Nine species were divided into groups I, II, and III based on their FA abundance. Data are mean ± SD with *n* = 3. Bars with no letters in common are significantly different (*p* < 0.01). **(B)** Dendrogram showing the association of nine species in clusters based on their FA content and composition.

**FIGURE 2:**
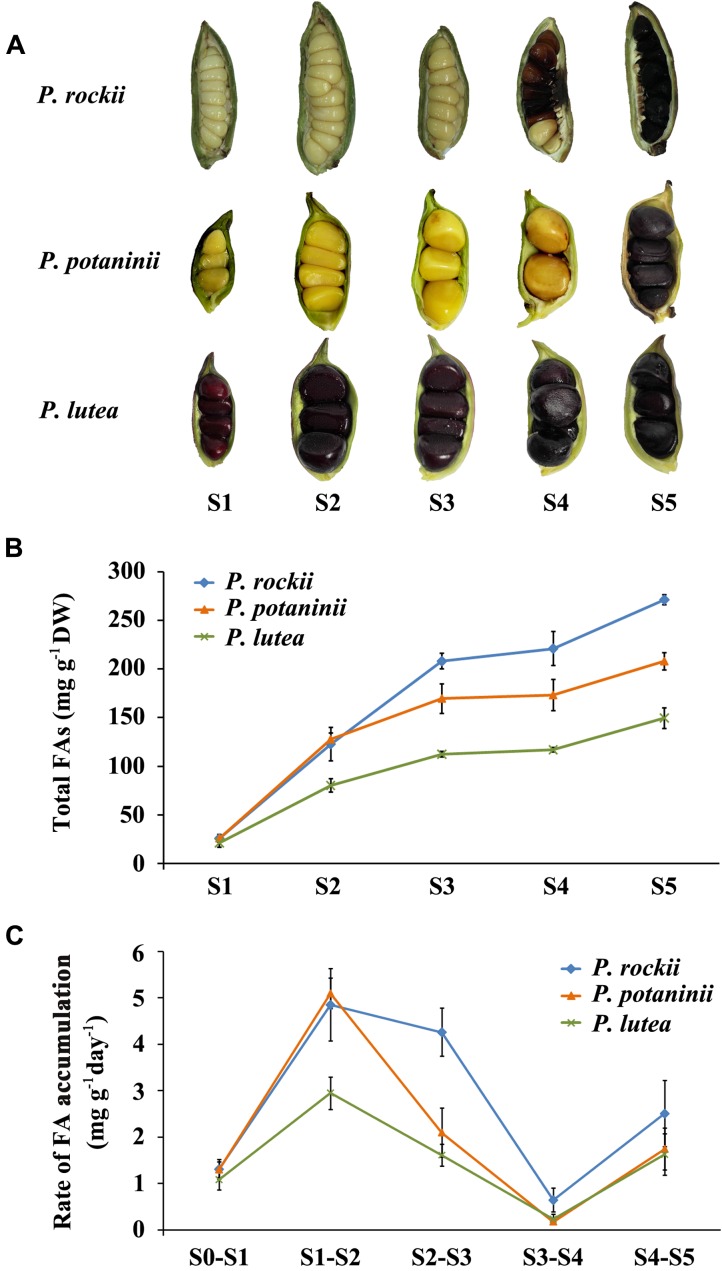
Phenotypic observation and fatty acid quantification of developing seeds of three peony species. **(A)** The developmental progress of three wild peony species seeds (S1–S5). Pods were harvested at 20 days after pollination (DAP, immature stage), and then every 20 days until 100 DAP (pods containing mature seeds). **(B)** Total fatty acids content was measured at five developmental stages during seed development of three wild peony species (mean ± SD, *n* = 3). **(C)** Rate of FA accumulation between stages was calculated per day (mean ± SD, *n* = 3).

**FIGURE 3:**
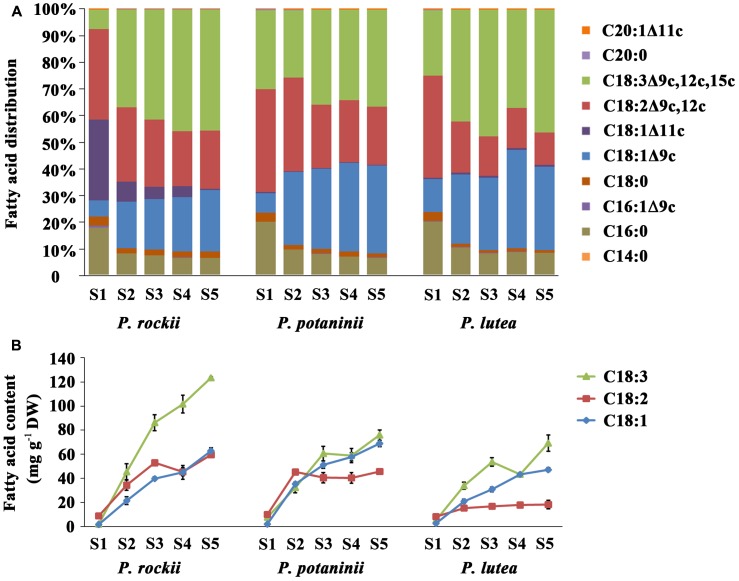
Fatty acid composition during seed development in three wild species. Changes in the **(A)** proportion of various FAs, and **(B)** content of three major unsaturated FAs during seed development (mean ± SD, *n* = 3).

**FIGURE 4:**
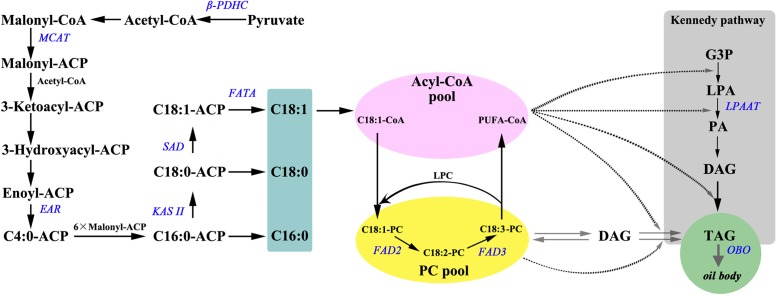
An overview of (A) major genes involved in fatty acid synthesis and triacylglycerol assembly. Substrates are in bold: ACP, acyl carrier protein; DAG, diacylglycerol; G3P, glycerol-3-phosphate; LPA, lyso-phosphatidic acid; LPC, lyso-phosphatidylcholine; PA, phosphatidic acid; PC, phosphatidylcholine; PUFA, polyunsaturated fatty acids; TAG, triacylglycerol. Enzymatic reactions are in bold italics: β*-PDHC*, pyruvate dehydrogenase beta subunit; *MCAAT*, malonyl-CoA:ACP transacylase; *EAR*, enoyl-ACP reductase; *KAS* II, ketoacyl-ACP synthase II; *SAD*, stearoyl-ACP desaturase; *FAD3*, Δ15 (ω-3) linoleic acid desaturase; *FATA*, acyl-ACP thioesterase A; *FAD2*, Δ12 oleic acid desaturase; *LPAAT*, 1-acylglycerol-3-phosphateacyltransferase; *OBO*, oil-body oleosin.

**FIGURE 5:**
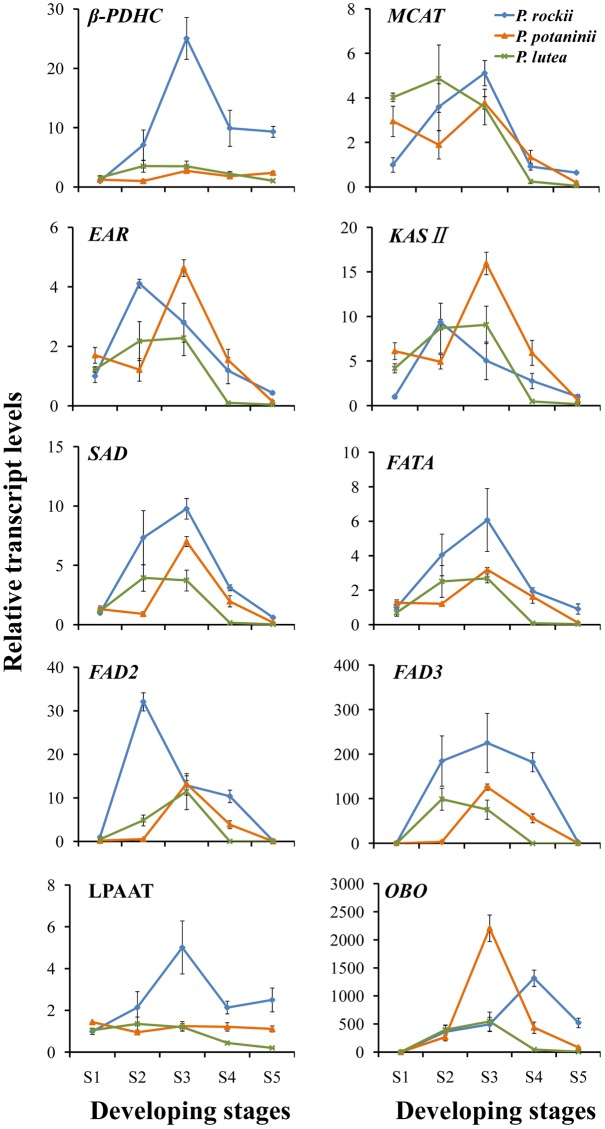
Quantitative real-time polymerase chain reaction analysis of genes involved in seed oil synthesis in three wild species at different developmental stages. Relative expression values, normalized to *26S-18S ITS* gene, were shown as 2^-ΔΔC_t_^ relative to 20 DAP of *P. rockii*. Error bars represent the SD of three biological replicates with three technical replicates each.

A lower ratio of ω-3 to ω-6 essential FAs has been implicated in chronic diseases in humans (Simopoulos, 2002; Harbige, 2003). Compared with traditional oil crops, tree peony seed oil, with its high rate of oil synthesis and 18:3 levels (∼45%) (Li et al., 2015a,b) contributes to nearly 5:3 ratio of ω-3 to ω-6 FAs (**Figures 1–3**), which represents a balanced source of PUFAs for human health and nutrition (Zhou et al., 2014). Our comparative study revealed subtle but key differences in rate and duration of oil accumulation (**Figures 1–3**) and associated gene expression (**Figures 4**, **5**) that might contribute to varying seed oil content in three tree peony species.

## Alpha-Linolenic Acid Is the Most Abundant FA Type in Matured Peony Seeds

Analysis of FA distribution during seed development of *P. rockii*, *P. potaninii*, and *P. lutea* (**Supplementary Table S2**) revealed that the three major unsaturated FAs (18:3, 18:2, and 18:1) contributed to about 90% of total content in stages 2–5 while it was about 76–78% in S1 (**Figure 3A**). ALA (36.3–46.0%) was the most predominant unsaturated FA followed by 18:2 (12.1–21.9%), and 18:1 (23.0–32.9%) in the mature stage (S5) of seed development (**Supplementary Table S2** and **Figure 3A**). Among the saturated FAs, 16:0 was the most predominant followed by 18:0. These five major FAs together account for 98.9% of total FA in mature seed (S5), the developmental stage at which FA content was at its highest (**Supplementary Table S2**). Five other minor FAs (<1.0%) including myristic acid (C14:0), palmitoleic acid (C16:1Δ9c), *cis*-11-octadecenoic acid (C18:1Δ11c), eicosanoic acid (C20:0) and *cis*-11-eicosenoic acid (C20:1Δ11c) were also detected at trace levels in mature tree peony seeds (**Supplementary Table S2**). In all the three species, total amount of 16:0 increased with seed development and was highest in matured seed (S5; **Supplementary Table S2**). This increase in 16:0 was, however, not as rapid as other unsaturated FAs and thereby the relative abundance of 16:0 in total FA acid content decreased from 20% in S1 to 10% in S5 (**Figure 3A**). The rapid increase in unsaturated 18C FAs during tree peony seed development, as supported by previous studies (Li et al., 2015a,b) suggests an active role for ketoacyl-ACP synthase II (KAS II) and 18:0-ACP desaturase (SAD). The relative proportions of 16:0 and 18:1 FAs, which are the main products of plastid FA synthesis (**Figure 4**) are also determined by the activities of acyl-ACP thioesterases (FAT) A and B (Bates et al., 2013).

### Nine Wild Tree Peony Species Exhibit Distinct FA Profile in Mature Seeds

Seed development process in wild peony species takes about 100 days from pollination to maturation at which time the pods break open and the seeds turn black in color. Developing seeds were harvested 20 days after pollination (DAP; stage 1, S1) and subsequently at 20-day intervals until maturation (stage 5, S5). Total FA content of mature seeds (at stage S5) of nine wild tree peonies varied significantly, ranging from 150 to 272 mg g^-1^ DW (**Figure 1A**). In all nine species, unsaturated FAs (18:3, 18:2, and 18:1) were most abundant, while saturated FAs (18:0 and 16:0) contributed to less than 10% of the total FA of matured seeds (**Supplementary Table S1**). FA content in seeds that were harvested at 10 days interval increased up to nine developmental stages and slightly decline in the last developmental stage (Li et al., 2015a). Despite the overall differences in FA content in mature stage, profile of dominant unsaturated (ALA, LA, OA) and saturated (PA, SA) FAs were consistent (Li et al., 2010, 2015a,b; Han et al., 2016; Yu et al., 2016). Cluster analysis, using the content of five major FAs in seeds of nine peony species (**Supplementary Table S1**) generated a dendrogram with three major clusters (**Figure 1B**). Species in cluster I, *P. rockii*, *P. decomposita*, and *P. qiui* were characterized by higher ALA, LA, and total FA content. Cluster II included *P. potaninii*, *P. ludlowii*, and *P. ostii*, with mid-level content of major FAs, while Cluster III included *P. jishanensis*, *P. lutea*, and *P. delavayi*, with lower levels of major FAs (**Supplementary Table S1**). Tree peony species within a cluster had a relatively similar range of FA content and composition suggesting that perhaps key steps in their FA biosynthesis and regulation are also conserved. To identify distinct biochemical and molecular differences that contribute to diverse levels of oil accumulation among closely related wild tree peony species, *P. rockii*, *P. potaninii*, and *P. lutea*, each representing a cluster with varying levels of FA content in mature seeds (**Figure 1**) were selected for further study.

### Rate of FA Accumulation during Seed Development Is Different among the Three Tree Peonies

Seeds from *P. rockii*, *P. potaninii*, and *P. lutea* varied in their size, color, total FA content, and rate of FA accumulation during seed development (**Figure 2**). As reported previously, mature seeds (S5) of all the three species were black in color while immature (S1) seeds varied from pale green in *P. rockii*, yellow in *P. potaninii*, to dark red in *P. lutea* (**Figure 2A**; Li et al., 2015a). Overall seed size was smaller with more seeds per fruit in *P. rockii*, relative to the other two species (**Figure 2A**). Quantification of FA content in the three species during seed development revealed differences not only in content and composition but also rate of lipid accumulation (**Supplementary Table S2** and **Figures 2B,C**). In all the three species, FA content peaked during seed maturation (S5; **Figure 2B**). A higher rate of FA accumulation was, however, observed only during the initial stages of seed development (S1 to S2) and not in the later stages (>S3; **Figure 2C**). During S1 to S2 development, among the three species, *P. rockii* (∼4.9 mg g^-1^ day^-1^) and *P. potaninii* (∼5.1 mg g^-1^ day^-1^) attained the highest rate of FA synthesis, while *P. lutea* showed the lowest (∼3.0 mg g^-1^ day^-1^; **Figure 2C**). Interestingly, while *P. rockii* maintained a higher rate of FA synthesis during S2 to S3 stages of seed development (∼4.3 mg g^-1^ day^-1^), both *P. potaninii* and *P. lutea* showed a 50% decline in their rate. As a general trend, however, in all the three species, the rate of FA accumulation declined as the seed development progressed from S2 to S4 but a slight increase was noted during S4 to S5 (**Figure 2C**). While reduced protein synthesis in terminal seed development period might contribute to increase in over all oil production, the rate and duration of oil accumulation are likely regulated by Wrinkled 1 (WRI1) transcription factor (Kanai et al., 2016). To determine such role for WRI1 in tree peony seeds would be pertinent in order to enhance oil accumulation. Over all these results suggest that early stages of seed development (S1–S3) are crucial periods for differential FA accumulation among the three wild peony seeds. These findings are also consistent with the previous study done by Li et al. (2015a), where FA accumulation in *P. ostii* was found to be rapid in early to mid stages of seed development rather than at maturation (Li et al., 2015a).

Further comparison of the temporal changes in FA content demonstrated that 18:3 is actively accumulated throughout the seed development and is the major contributor to seed oil synthesis in these select wild peony species (**Figure 3B**). Among the three, *P. rockii* had highest 18:3 content, which rapidly increased from S1 to S5, while an increase in 18:2 and 18:1 was relatively moderate and peaked in S3 (**Figure 3B**). In *P. potaninii* seeds, while 18:1 and 18:3 increased till maturation, 18:2 reached its highest level at S2. In seeds of *P. lutea*, although 18:2 did not vary with development, 18:3 and 18:1 continued to accumulate at a relatively slow rate (**Figure 3B**). These data together indicate that *P. rockii*, relative to other peonies, perhaps has mechanisms in place for rapid accumulation of FAs, specifically 18:3. These data also suggest that 20–60 DAP, the period during which fruits of tree peony expand rapidly as a key period for differential rate of FA accumulation that determines the final FA content and composition of mature seeds.

### Gene Expression Analysis Reveals Differential Transcript Abundance among the Tree Peony Species

Based on the previous studies (Fatima et al., 2012; Wang et al., 2012; Zhou et al., 2014), 10 lipid biosynthesis genes (**Figure 4**) were selected to determine if their expression pattern during seed development correlated with changes in lipid content and composition (**Supplementary Table S2**). Nine of the 10 select genes (β*-PDHC*, *MCAT*, *EAR*, *KAS* II, *SAD*, *FATA*, *FAD2*, *FAD3*, and *LPAAT*) encode for enzymes that play a major role in FA synthesis and TAG assembly, while oleosin (OBO) is essential for packaging of TAG into oil bodies in seed tissues (**Figure 4**). Since multiple genes encode several of the proteins involved in oil biosynthesis, we relied on transcriptome data from a previous study on *P. ostii* to select those unigenes that were upregulated by several fold during oil accumulation period in tree peony seeds (Li et al., 2015a). In all the three species, expression levels for β*-PDHC*, *MCAT*, *EAR*, *KASII*, *SAD*, and *FATA* peaked within 60 DAP (during S1–S3; **Figure 5** and **Supplementary Figure S1** and **Supplementary Table S4**) suggesting that an active *de novo* FA synthesis might be occurring during early to mid developmental stages of tree peony seeds, which is typical of several other oilseeds (Troncoso-Ponce et al., 2011). These peak expression levels at S3 in *P. rockii*, relative to its S1 were 25-fold higher for β*-PDHC* and 5-fold higher for *MCAT* (**Supplementary Figure S1** and **Supplementary Table S4**), genes that encode for crucial precursors acetyl-CoA and malonyl-ACP, respectively, for FA synthesis. In *P. rockii*, expression levels for KAS II and SAD were also more than ninefold higher in their peak stage, S2 and S3, respectively, relative to S1, which correlates with abundance of 16:0 in early developmental stage followed by increase in 18:1 at later stages (**Figure 3A**). Such increase remained below twofold in *P. potaninii* and *P. lutea* (**Supplementary Figure S1** and **Supplementary Table S4**), suggesting a correlation between the plastidial FA gene expression levels and oil accumulation. Furthermore, expression levels for most plastidial FA genes remained higher or similar to the levels at S-1 during the entire seed development period in *P. rockii* but reduced below S1 levels by 80 DAP in *P. potaninii* and even earlier in *P. lutea* (**Supplementary Figure S1** and **Figure 5**), suggesting that the duration of transcriptional activity might affect overall oil accumulation and thus contribute to differential oil content in matured tree peony seeds (Kanai et al., 2016).

Triacylglycerol assembly via DGAT catalyzed acyl-CoA-dependent or a PC-derived pathway (**Figure 4**) utilizes PC as a central intermediate in maintaining the flux of FAs and/or DAG (Ohlrogge and Browse, 1995; Bates and Browse, 2012). Since the *sn*-2 position of PC is the major site for ER localized FA modification such as desaturation, and hydroxylation (Sperling et al., 1993; van de Loo et al., 1995), understanding the acyl flux into and out of PC is crucial for improving the production of PUFA (18:3)-enriched TAG (Wallis et al., 2002). The expression of *FAD3* in *P. rockii* was characterized by a bell-shaped curve, with low levels of expression at the initial stage followed by a substantial increase during the rapid phase of oil accumulation and a subsequent decline toward seed maturation (**Figure 5** and **Supplementary Figure S1**), a conserved pattern that was previously observed in oil-rich Arabidopsis, *Brassica napus* and sea buckthorn seeds (Hu et al., 2009; Peng and Weselake, 2011; Fatima et al., 2012). Together, the expression pattern and transcript abundance of *FAD3* and *FAD2*, with *FAD3* being much higher than *FAD2* in *P. rockii* suggest that its high 18:3 content might be the result of *FAD3*-dependent active PC-derived pathway (Bates et al., 2013). Expression of *FAD3* in perilla and sacha inchi seeds also coincided with the accumulation of 45–53% of total FA as ALA (Chung et al., 1999; Wang et al., 2012), further suggesting FAD3 as a valuable target for genetic engineering and improving ALA content in oil crops. On the other hand, relatively low expression levels of *FAD2* and *FAD3* in seeds of *P. lutea* (**Figure 5**) might account for its lower proportion of LA and ALA content (**Figure 3**). Transcript levels for *LPAAT*, which with its high substrate specificity determines acyl composition of TAG at the *sn*-2 position (Baud and Lepiniec, 2010), were fivefold higher in *P. rockii* at 60 DAP relative to 20 DAP (**Supplementary Table S4**). In *P. rockii* seeds, transcripts for *OBO*, as expected, peaked in S4, toward the end of FA synthesis phase as needed for packaging TAG (**Figure 5** and **Supplementary Figure S1**). Interestingly *OBO* expression levels for *P. potaninii* peaked by 60 DAP and were higher than that of *P. rockii*. Oleosins typically determine the size of the oil bodies (Siloto et al., 2006) and it remains to be determined if there is variation in oil body size in tree peony seeds.

Temporal changes in the expression pattern of these 10 genes during seed development, relative to the transcript levels in S1 of *P. rockii* also revealed highest expression levels mostly during S2–S3 stages in *P. rockii* and *P. lutea*, and S3 stage of *P. potaninii* (**Figure 5**). These stages with higher transcript abundance also correlated with the stages during which higher rate of FA accumulation (**Figure 2C**). Conversely, reduced transcript abundance was observed during initial (S1) and late (S4 and S5) developmental stages, coinciding with the periods of low rate of FA accumulation (**Figure 2C**). Specifically, a three–fourfold higher expression level for β*-PDHC*, *FAD2*, *FAD3*, *LPAAT*, and *OBO* in *P. rockii* were noted relative to *P. lutea* (**Figure 5**), which was in agreement with its higher total FA and ALA content (**Figures 2**, **3**). Transcript abundance for *MCAT*, *EAR*, *KAS* II, *SAD*, and *FATA* differed by less than twofold between the species suggesting these genes might not play a significant role in differential FA accumulation in developing seeds of peonies (**Figure 5**).

## Conclusion

Alpha-linolenic acid cannot be synthesized by the human body (Sinclair et al., 2002; Liu et al., 2012), and yet is an essential precursor for the synthesis of eicosapentaenoic acid and docosahexaenoic acid, which exert a wide range of biological activities and prophylactic effects (Shahidi and Miraliakbari, 2004; Shahidi and Miraliakbari, 2005). Considering the increasing population (Chapman and Ohlrogge, 2012) there is a need for the development of oil crops with beneficial proportions of ω-3 and ω-6 FAs. In this study, we conducted comparative FA and gene expression analyses of developing seeds of three peony species with differences in the rate, content and composition of FAs to identify fundamental determinants of FA content and composition. Our results indicate that a higher and sustained lipid gene expression levels in *P. rockii* might contribute to its increased rate and duration of TAG accumulation and thus to an overall increase in seed oil content. Furthermore, although the expression pattern of all the ten genes do not correlate with the pattern of oil accumulation, the data together suggest that *P. rockii* has a more efficient metabolic pathway to synthesize ALA compared with *P. potaninii* and *P. lutea*, which is likely due to the abundance of *FAD3* transcripts in the PC-derived pathway. Although transcript levels do not necessarily reflect protein or its activity, the temporal expression patterns for *FAD2* and *FAD3* associated with PUFA-enriched FA profile suggests an important role for them in determining the FA composition in peony seeds. These results also revealed a significant role for β*-PDHC*, *LPAAT*, and oleosin since their high expression level was in agreement with the highest total FA content in *P. rockii*. An increase in oil content even after the decline in transcript levels might suggest involvement of additional genes that might also play an important role in PUFA synthesis. In conclusion, however, *FAD3, FAD2*, β*-PDHC*, *LPAAT*, and oleosin were identified as potential targets for molecular cloning and functional characterization and to further improve oil content and composition in tree peonies as well as other crops. Among the tree peonies, *P. rockii* is an excellent germplasm resource for cultivating high yielding and high quality peony oil and could be of further improved with identification of molecular markers and using transgenic approaches.

## Author Contributions

Q-YZ, L-XN, and Y-LZ designed the research. Q-YZ, RY, and L-HX harvested the samples, and conducted the lipid and gene expression analyses. Q-YZ, MR, AK, and Y-LZ conducted the data analyses and wrote the manuscript. All authors read and approved the final manuscript.

## Conflict of Interest Statement

The authors declare that the research was conducted in the absence of any commercial or financial relationships that could be construed as a potential conflict of interest.

## Supplementary Material

FIGURE S1 Gene expression profiles for developing seeds, relative to their S1 for the three tree peony species.

TABLE S1 Content of the five major FAs in matured seeds (stage S5) of nine tree peony species (mg g^-1^ DW, mean ± SD, *n* = 3).

TABLE S2 Fatty acid composition in three wild relatives of tree peony during seed development (mg g^-1^ DW, mean ± SD, *n* = 3).

TABLE S3 Gene-specific primer sequences for detection by qRT-PCR.

TABLE S4 Transcript levels of 10 genes in developing seeds of three tree peonies, relative to their S1 **(A)** and S1 of *P. rockii* **(B)**. Data with conditional formatting reveal the highest expression for each gene in red and lowest in green.

## Abbreviations

ALA: α-linolenic acid
DAG: diacylglycerol
DAP: days after pollination
DGAT: DAG acyltransferase
DW: dry weight
ER: endoplasmic reticulum
FA: fatty acid
FAD: fatty acid desaturase
FAMEs: fatty acid methyl esters
G3P: glycerol-3-phosphate
GC-MS: gas chromatography-mass spectrometry
GPAT: G3P acyltransferase
LA: linoleic acid
LD: Lipid droplet
LPAAT: lysophosphatidic acid acyltransferase
PAP: phosphatidic acid phosphatase
PC: phosphatidylcholine
PUFA: polyunsaturated fatty acid
qRT-PCR: quantitative real-time polymerase chain reaction
TAG: triacylglycerol
