# Supplementary figures and images for: Fatty Acid and Associated Gene Expression Analyses of Three Tree Peony Species Reveal Key Genes for α-Linolenic Acid Synthesis in Seeds
Source: Front Plant Sci. 2018 Feb 5;9:106. doi: 10.3389/fpls.2018.00106 (PMC5807371; doi:10.3389/fpls.2018.00106)

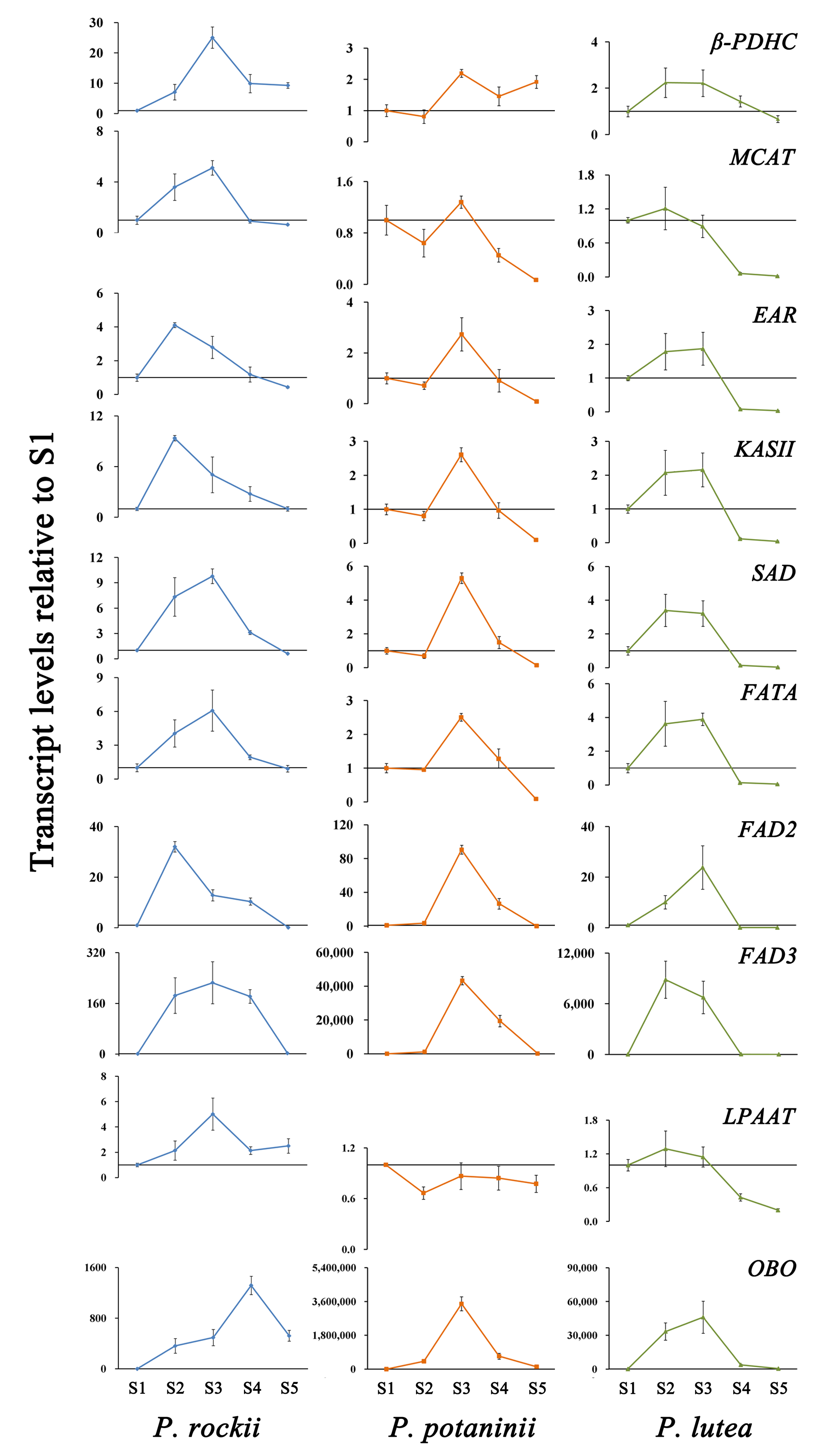

Supplement: FIGURE S1 — Gene expression profiles for developing seeds, relative to their S1 for the three tree peony species. [file Image_1.TIF]
